# Vaccination and the risk of systemic lupus erythematosus: a meta-analysis of observational studies

**DOI:** 10.1186/s13075-024-03296-8

**Published:** 2024-03-04

**Authors:** Meijiao Wang, Huanpeng Gu, Yingqi Zhai, Xuanlin Li, Lin Huang, Haichang Li, Zhijun Xie, Chengping Wen

**Affiliations:** Research Institute of Chinese Medicine Clinical Foundation and Immunology, School of Basic Medicine Sciences, Zhejiang Chinese Medical University, Binwen Road, Binjiang Dsitrict, Hangzhou, China

**Keywords:** Vaccination, Systemic lupus erythematosus, Risk, Meta-analysis

## Abstract

**Objective:**

This meta-analysis aims to explore the potential link between vaccines and systemic lupus erythematosus (SLE).

**Methods:**

We systematically searched PubMed, Cochrane Library, and Embase for observational studies from inception to September 3, 2023, using medical subject headings (MeSH) and keywords. Study quality was assessed using the NOS scale. Statistical analyses were conducted using STATA software (version 14.0). Publication bias was evaluated using funnel plots and Egger’s regression.

**Results:**

The meta-analysis incorporated 17 studies, encompassing 45,067,349 individuals with follow-up periods ranging from 0.5 to 2 years. The pooled analysis revealed no significant association between vaccinations and an increased risk of SLE [OR = 1.14, 95% CI (0.86–1.52), I^2^ = 78.1%, *P* = 0.348]. Subgroup analyses indicated that HBV vaccination was significantly associated with an elevated risk of SLE [OR =2.11, 95% CI (1.11-4.00), I^2^ = 63.3%, *P* = 0.02], HPV vaccination was slightly associated with an increased risk of SLE [OR = 1.43, 95% CI (0.88–2.31), I^2^ = 72.4%, *P* = 0.148], influenza vaccination showed no association with an increased risk of SLE [OR = 0.96, 95% CI (0.82–1.12), I^2^ = 0.0%, *P* = 0.559], and COVID-19 vaccine was marginally associated with a decreased risk of SLE [OR = 0.44, 95% CI (0.18–1.21), I^2^ = 91.3%, *P* = 0.118].

**Conclusions:**

This study suggests that vaccinations are not linked to an increased risk of SLE. Our meta-analysis results provide valuable insights, alleviating concerns about SLE risk post-vaccination and supporting further vaccine development efforts.

**Supplementary Information:**

The online version contains supplementary material available at 10.1186/s13075-024-03296-8.

## Introduction

Systemic lupus erythematosus (SLE) is a prototypical autoimmune disease that affects multiple body systems and organs. It is characterized by loss of immune tolerance, sustained production of autoantibodies, hyperactive B cell and T cell responses and the breakdown of immune tolerance towards self-antigens [1, 2]. While the exact etiology of SLE remains incompletely understood, a complex interplay of genetic predisposition and environmental triggers is believed to be at the core. Notably, infectious agents, predominantly viruses, have been implicated in the initiation or exacerbation of SLE [3, 4]. Patients with SLE often experience immunosuppression, either spontaneously or as a consequence of treatment, rendering them more susceptible to infections [5, 6]. Hence, there is a compelling need for preventative vaccination.

Vaccines play an indispensable role in modern medicine and are meticulously designed to confer protection against viral, bacterial, or fungal infections in host organisms. Typically administered prophylactically, vaccines are a standard practice in safeguarding the health of the general population (children, adolescents and adults in good health) [7]. However, concerns regarding the potential link between infections and autoimmunity, as well as the risk of precipitating or exacerbating lupus following vaccination, have led to hesitancy in offering vaccinations to individuals with SLE [8, 9]. Vaccinations have been theorized as potential triggers for the onset of SLE, considering their role in eliciting antigen-specific immune responses [10].

It was reported an equivalent occurrence of autoimmune disorders in vaccinated and unvaccinated individuals, absence of clear association between HPV, HBV vaccines and autoimmune diseases, and the benefits of COVID-19 vaccination far outweigh the possible new-onset autoimmune diseases [11–14]. While an earlier review suggested a connection between vaccinations and an elevated risk of SLE [15], subsequent studies have yielded conflicting results, with some demonstrating no discernible impact on SLE risk [16–19]. Due to these contradictory findings, a definitive conclusion regarding the relationship between vaccinations and the risk of SLE has remained elusive. To gain a more comprehensive understanding of the association between vaccinations and the risk of SLE, we conducted this meta-analysis.

## Methods

The meta-analysis was conducted in accordance with the guidelines of the Preferred Reporting Items for Systematic Reviews and Meta-Analyses (PRISMA) [20]. The protocol was pre-registered in the International Prospective Register of Systematic Reviews (PROSPERO) platform, and the approval number is CRD42023460701.

### Data sources

PubMed, Cochrane Library, and Embase were searched for studies published from database inception to September 3, 2023. There were no language restrictions, and the search strategy combined the use of Medical Subject Headings (MeSH) and keywords. The search terms included systemic lupus erythematosus, SLE, vaccination, and their variants. The details of the search strategy were shown in supplementary Tables 1–3.

### Eligibility criteria

The observational studies were included on the basis of the following criteria: (1) cohort studies or case-control studies; (2) investigations of the association of vaccination and the risk of SLE. In this meta-analysis, any type of vaccine was included. Observational studies were excluded if they did not provide an odds ratio (OR) with corresponding 95% confidence interval (CI). If more than one study reported data from the same study, we included the study with the longest follow-up or the largest number of participants. This meta-analysis also excluded conference abstracts, study protocols, letter to editor, duplicate publications, and studies with no outcomes of interest.

### Study selection

Study selection was performed by two reviewers (MJ Wang and HP Gu) who independently screened the literature based on the eligibility and exclusion criteria. Duplicate and irrelevant articles were first excluded according to their titles and abstracts. After that, the full texts of the potentially eligible articles were downloaded and read to identify all eligible studies. Any disagreements were resolved by the third reviewer (XL Li), who acted as an arbiter.

### Data extraction

Data extraction was performed independently by the two above-mentioned reviewers (MJ Wang and HP Gu) who consulted he guidelines on data extraction for systematic reviews and meta-analysis [21]. We used predesigned forms for extracting data including the first author, year of publication, study type, sample size, follow-up years, age, diagnosis of SLE, vaccine type, observation time, and observation region. Disagreements were resolved by discussion with XL Li to reach a consensus.

### Risk of bias

The Newcastle-Ottawa scale (NOS) was used to assess the study quality [22]. NOS include three aspects: selection, comparability, and exposure. Stars ranged from 0 to 9 points for the studies, four stars for selection of participants and measurement of exposure, two stars for comparability, and three stars for assessment of outcomes and adequacy of follow-up, with more stars indicating higher quality of study. Scores of 0–3, 4–6, and 7–9 were considered to indicated low, moderate, and high quality, respectively.

### Evidence certainty

The Grading of Recommendations Assessment, Development, and Evaluation (GRADE) system was employed to access the overall certainty of evidence [23, 24]. By the GRADE system, the certainty of evidence derived from observational studies receives an initial grade of low quality. The quality of evidence from cohort studies can be improved at larger effect sizes (RR ≥ 2 or ≤ 0.5), dose-response gradients, or attenuation by plausible confounding after excluding various factors that could lead to downgrading [25]. Finally, the evidence of outcomes can be graded as high, moderate, low, or very low [26].

### Statistical analysis

The adjusted odds ratios (OR) and their corresponding 95% confidence intervals (CI) from each trial were utilized to evaluate the relationship between vaccination and the risk of SLE. Heterogeneity was assessed using the χ^2^-test and I^2^-values. A fixed-effects model was applied when the P-value exceeded 0.1, and the I^2^ statistic was less than 50%. In cases where I^2^ surpassed 50%, signifying substantial heterogeneity, a random-effects model was employed. To ensure the robustness of the overall results, a sensitivity analysis was conducted. Publication bias was evaluated by visually inspecting the funnel plot and statistically testing it using Egger’s regression. Subgroup analyses were carried out based on geographical region, length of observation, and study design. All statistical analyses were conducted using Stata statistical software version 14.0, developed by Stata Corp in College Station, Texas, USA, for added precision and reliability.

## Results

### Literature search

**Fig. 1:**
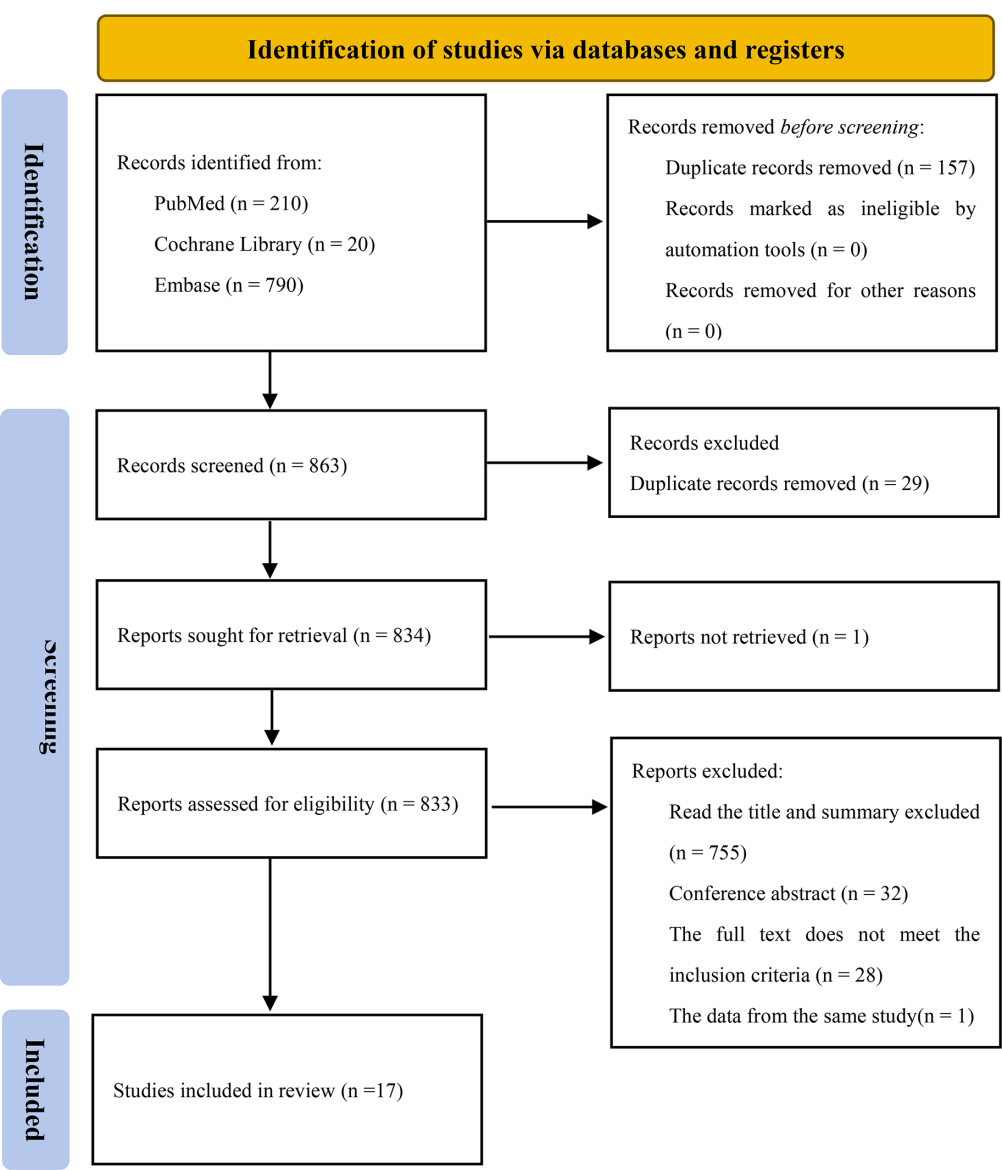
Studies screening process

A total of 1020 records were collected through the search. After title and abstract screening 46 articles were considered potentially relevant. Seventeen studies were included after full text review, which reported the incidence of SLE on follow-up. The selection process is presented in Fig. 1.

### Study characteristics

This meta-analysis encompassed 17 studies, enrolling a total of 45,067,349 individuals, with a publication date up to September 3, 2023. Of these studies, ten were cohort investigations, and the remaining seven were case-control studies. The average follow-up duration across these studies varied from 0.5 to 2 years, and most studies consistently adhered to well-defined diagnostic criteria for SLE. While the adjusted estimates were available for nearly all studies, it’s worth noting that there were slight variations in the adjusted confounders employed. The primary characteristics of the studies included in this analysis are detailed in Table 1, and the excluded studies were shown in supplementary Table 4.

**Table 1:** Characteristics of the included studies

Author	Year	Country	Study type	Study size	Follow-up	Age (years)	Observation time	Diagnosis of SLE	Vaccine type	NOS scores
Ju HJ [16]	2023	Korea	Cohort study	3,838,120 vaccination, 3,834,804 historical	(100.7 ± 90.3), (100.7 ± 88.5) days	(45.7 ± 18.7), (44.8 ± 18.7)	NA	ICD-10	COVID-19	5
Peng K [27]	2023	Hong Kong	Cohort study	4,197,188 population	NA	54.09 ± 18.08, 54.25 ± 18.33	28 days	ICD-9	COVID-19	7
Skufca J [17]	2018	Finland	Cohort study	134,615 vaccinated, 105,990 unvaccinated	365 days	(11–15)	NA	ICD-10	HPV	6
Hviid A [18]	2018	Danish and Swedish	Cohort study	319,298 vaccinated, 16,067,162 unvaccinated	NA	18–44	NA	ICD-10	HPV	5
Miranda S [19]	2017	French	Cohort study	1,393,167 vaccination, 4,746,593 unvaccinated	NA	13–16	(20 ± 11) months	ICD-10	HPV	5
Geier DA [28]	2017	USA	Case-control study	48,816 cases, 21,998 controls	3–37 days	6–39	NA	SLE (VAERS code: 10,042,945)	HPV	6
Bardenheier BH [29]	2016	USA	Case-control study	39 cases,117 controls	1 year	most in the 18- to 24- year-old range	3 years	ICD-9	AVA	7
Lai YC [30]	2015	USA	Case-control study	80 cases, 151 controls	NA	NA	NA	SLE (VAERS code: 10,042,945)	Zoster	5
Grimaldi-Bensouda L [31]	2014	France and Canada	Case-control study	105 cases, 712 controls	24 months	(32.9 ± 12.6), (34.8 ± 13.8)	12 or 24 months	The 1997 ACR criteria	Any vaccine (including influenza, DTPP)	7
Persson I [32]	2014	Sweden	Cohort study	3,347,467 vaccination, 2,497,572 unvaccination	27 months	1->80	45 days	ICD-10	Influenza	9
Zou Y [33]	2014	China	Case-control study	471 cases identified from 1,253,832 individuals	NA	(39.08 ± 11.03), (39.05 ± 10.96)	NA	The 1997 ACR criteria	HBV	7
Angelo MG [34]	2014	40 countries	Cohort study	31,173 HPV, 24,241 controls	NA	8–72	30 days (day 0–29)	NA	HPV	6
Arnheim-Dahlström L [35]	2013	Denmark, Sweden	Cohort study	230,005 vaccinated, 2,374,231 unvaccinated	180 days	12.8 (2.7)	NA	ICD-10	HPV	8
Chao C [36]	2012	USA	Cohort study	117,761 vaccinated, 412,151 unvaccinated	180 days	(9–26)	NA	ICD-9	HPV	6
Verstraeten T [37]	2008	USA	Cohort study	36,744 cases, 31,768 controls	1.8 years	10–72	NA	NA	HPV, HSV, HBV	6
GEIER DA [38]	2005	USA	Case-control study	47 cases,782 controls	NA	24–39	(3–19) days	Costart Code	HBV	6
Cooper GS [39]	2002	USA	Case-control study	265 cases, 355 controls	NA	15–81	13 months	The 1997 ACR criteria	HBV	7

(ICD: international classification of diseases, COVID-19: coronavirus disease 2019, HPV: human papillomavirus, HBV: hepatitis B virus, HSV: herpes simplex virus, NA: not applicable, DTPP: diphtheria, tetanus, pertussis, poliomyelitis)

### Quality assessment

According to NOS criteria, the average score was 6.35 of all included observational studies, and the score for each trail was 5 or above, indicating that all studies were of moderate, or high quality in this meta-analysis. The scores of the included studies are shown in Table 1. The details were specified in supplementary Table 5.

### The different type of vaccine and the risk of SLE

There were seven studies of human papillomavirus (HPV) vaccine [17–19, 28, 34–36], four studies of hepatitis B virus (HBV) vaccine [33, 37–39], two influenza vaccine [31, 32], two coronavirus disease 2019 (COVID-19) vaccine [16, 27], one zoster vaccine [30], one AVA vaccine [29], one mentioned HPV, HSV and HBV vaccine [37], one mentioned any vaccines (including influenza, DTPP) [31], and one no mentioned the type of vaccine [39] explored the association between a history of vaccination and the risk of SLE [31, 37, 39]. The pooling analysis showed that a history of HBV vaccination is significant associated with an increased risk of SLE [OR = 2.11, 95% CI (1.11-4.00), I^2^ = 63.3%, *P* = 0.02], HPV vaccination was slightly associated with an increased risk of SLE [OR = 1.43, 95% CI (0.88–2.31), I^2^ = 72.4%, *P* = 0.148], influenza vaccination was no associated with an increased risk of SLE [OR = 0.96, 95% CI (0.82–1.12), I^2^ = 0.0%, *P* = 0.559], COVID-19 vaccine marginally significant associated with an decreased risk of SLE [OR = 0.44, 95% CI (0.18–1.21), I^2^ = 91.3%, *P* = 0.118].

### Any vaccine and risk of SLE

**Fig. 2:**
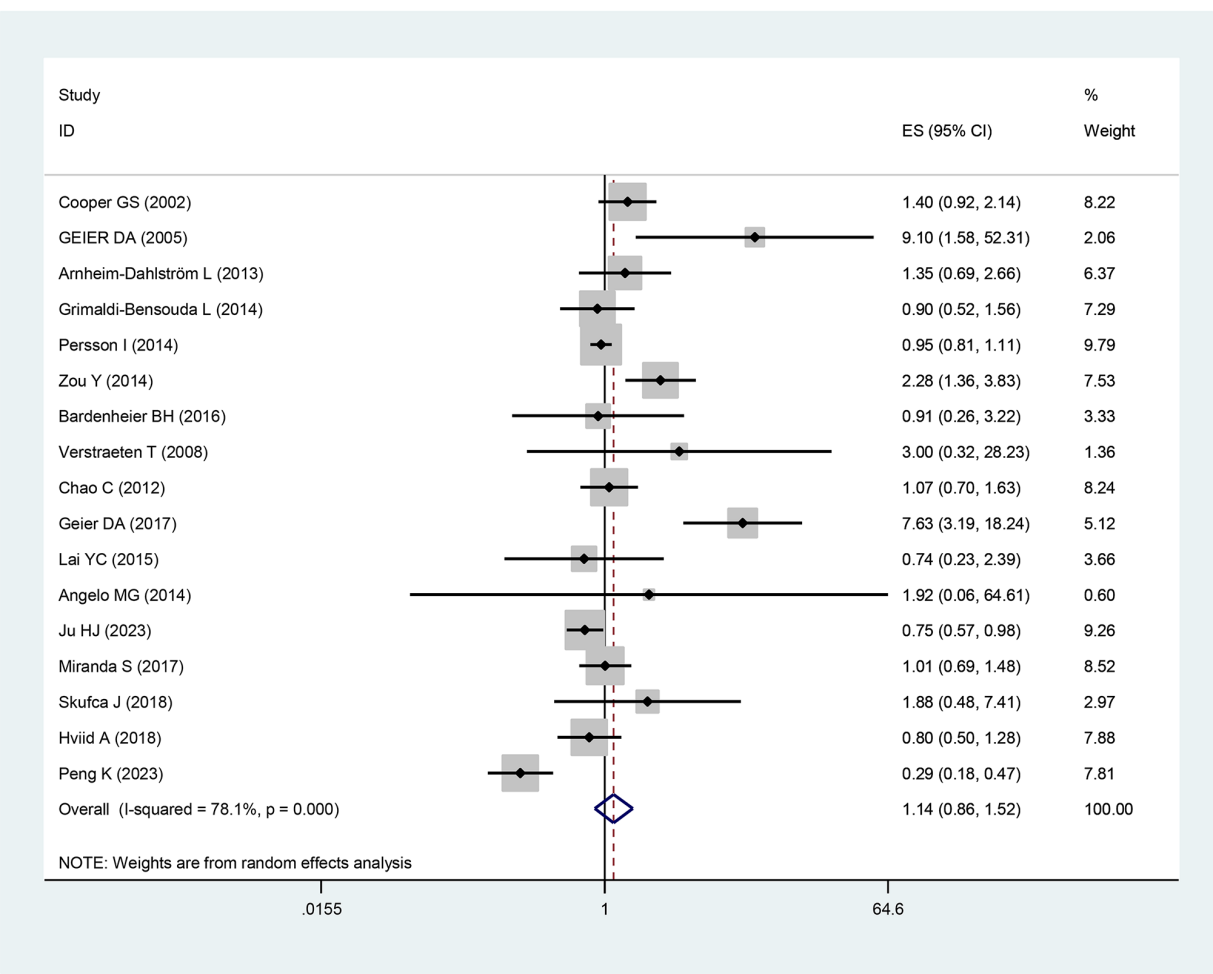
Meta-analysis of the risk of SLE associated with any vaccine

Seventeen studies explored the association between a history of vaccination and the risk of SLE. The pooling analysis showed that a history of vaccinations was not markedly associated with an increased risk of SLE [OR = 1.14, 95% CI (0.86–1.52), I^2^ = 78.1%, *P* = 0.348, Fig. 2]. Sensitivity analysis showed that none of the individual studies reversed the pooled-effect size, which means that the results are robust (supplementary Fig. S1, and supplementary Table 6).

### Subgroup analysis

In the subgroup analysis, a history of vaccination in North America, Europe and Asia suggested a marginally significant association between vaccinations and risk of SLE [OR = 1.87, 95% CI (0.99–3.52), I^2^ = 73.2%, *P* = 0.053; OR = 0.96, 95% CI (0.84–1.10), I^2^ = 0.0%, *P* = 0.551; and OR = 0.79, 95% CI (0.30–2.07), I^2^ = 93.9%, *P* = 0.628, respectively] (Table 2). There was no significant association between vaccination and the risk of SLE at any of length of observations (Table 2). In the subgroup analyses by study design, meta-analysis of case-control studies showed a significant association between vaccinations and increased risk of SLE [OR = 1.84, 95% CI (1.03–3.31), I^2^ = 75.9%, *P* = 0.041], whereas meta-analysis of cohort studies suggested a slightly significant association between vaccinations and risk of SLE [OR = 0.85, 95% CI (0.65–1.11), I^2^ = 68.3%, *P* = 0.243] (Table 2).

**Table 2:** Subgroup analysis for the risk of vaccination with SLE

Subgroups	Included studies	OR (95% CI)	Heterogeneity	
			I ^2^ (%)	P-value
Region				
North America	7	1.87 (0.99, 3.52)	73.2	0.053
Europe	7	0.96 (0.84, 1.10)	0.0	0.551
Asia	3	0.79 (0.30, 2.07)	93.9	0.628
Observation time				
≥ 180 days	8	1.09 (0.89, 1.33)	0.0	0.408
< 180 days	8	1.13 (0.68, 1.87)	87.3	0.634
Study type				
Case-control study	7	1.84 (1.03, 3.31)	75.9	0.041
Cohort study	10	0.85 (0.65, 1.11)	68.3	0.243

### Evidence certainty

The GRADE level of evidence is very low for the risk of SLE with any vaccine. The GRADE level of evidence is very low for the risk of SLE with HPV vaccine and HBV vaccine, and is low for the risk of SLE with influenza, and COVID-19 vaccine. The GRADE level of evidence is very low for the risk of SLE in case-control study and cohort study. The GRADE level of evidence is very low for the risk of SLE in North America and Asia, and is low for the risk of SLE in Europe. The GRADE level of evidence is low for the risk of SLE in observation time ≥ 180 days, and is very low for the risk of SLE in observation time < 180 days. GRADE evidence certainty for the outcomes is shown in Table 3.

**Table 3:** GRADE certainty of evidence

Outcome	Exposure	Study numbers	GRADE					Evidence quality
			Risk of bias	Inconsistency	Indirectness	Imprecision	Publication bias	
SLE	Any vaccine	17	0	-1^a^	0	0	0	Very Low
SLE	HPV vaccine	7	0	-1^a^	0	0	0	Very Low
SLE	HBV vaccine	4	0	-1^a^	0	0	0	Very Low
SLE	Influenza vaccine	2	0	0	0	0	0	Low
SLE	COVID-19 vaccine	2	0	-1^a^	0	0	0	Low
SLE	Case-control study	7	0	-1^a^	0	0	0	Very Low
SLE	Cohort study	10	0	-1^a^	0	0	0	Very Low
SLE	North America	7	0	-1^a^	0	0	0	Very Low
SLE	Europe	7	0	0	0	0	0	Low
SLE	Asia	3	0	-1^a^	0	0	0	Very Low
SLE	Observation time ≥ 180 days	8	0	0	0	0	0	Low
SLE	Observation time < 180 days	8	0	-1^a^	0	0	0	Very Low

SLE: systemic lupus erythematosus, Explanations: a. high heterogeneity

### Publication bias

**Fig. 3:**
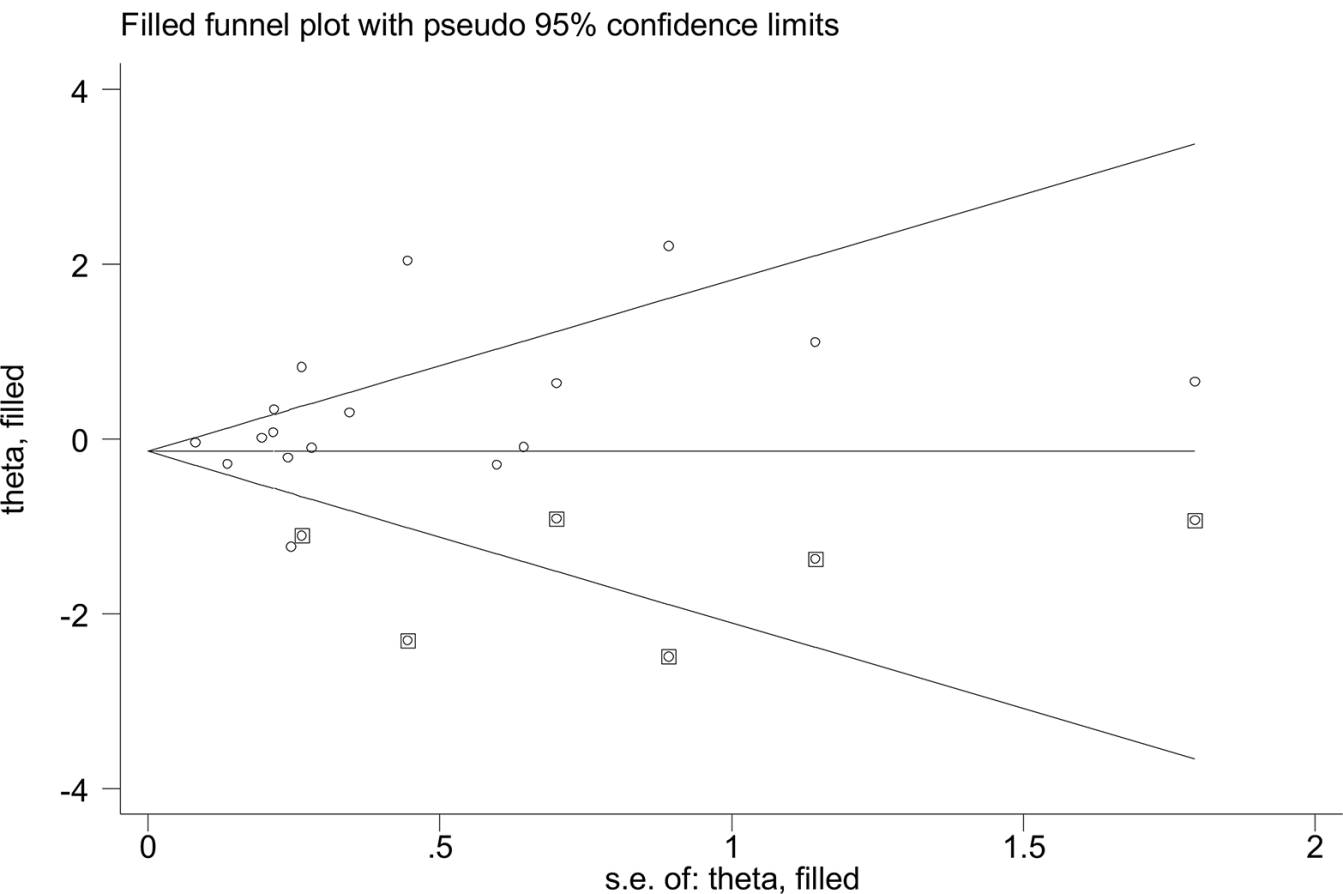
Publication bias of the risk of SLE associated with any vaccine

A visual inspection of the funnel plot showed a significant publication bias in the outcome of vaccination and risk of SLE. Correction was implemented using the trim and fill method. After inferring possible missing studies, the corrected odds ratio for vaccination associated with the risk of SLE was found to be 0.87 (90% CI 0.64–1.18, *P* = 0.361, Fig. 3), indicating vaccination is not associated with increased risk of SLE. The trim and fill method demonstrated a roughly symmetrical distribution of the funnel plot, thus exhibiting less susceptibility to publication bias and greater credibility.

## Discussion

### Main findings

Our meta-analysis encompassed 17 studies involving 45,067,349 individuals, providing a comprehensive assessment of the relationship between vaccinations and risk of SLE. We observed no significant increase in the risk of SLE among those who were vaccinated compared to non-vaccinated controls, suggesting that vaccination may not be an independent risk factor for SLE. Subgroup analyses, including history of HPV, influenza and COVID-19 vaccination, vaccination in North American, Europe and Asia, and cohort studies, revealed no substantial association between vaccinations and an increased risk of SLE. However, the meta-analysis of HBV vaccination, and case-control studies indicated a significant connection between vaccinations and risk of SLE, respectively.

### Interpretation of findings

A previous review included 12 studies investigated those vaccinations significantly increased the risk of SLE [15]. The result was still consistent at short vaccinated time, excluding high risk of bias studies, studies without receiving funds from pharmacemeutical companies, and outcomes of short vaccinated. In the subgroup analyses, showed a significant association between vaccinations and increased risk of SLE in case-control studies, while suggested a marginally significant association between vaccinations and increased risk of SLE in cohort studies. In the included 12 studies, there were five studies mentioned HPV vaccine [28, 34–37], two of influenza vaccine [31, 32], and four mentioned HBV vaccination [33, 37–39], and subgroup analyses showed HBV vaccination was significantly linked to an increased risk of SLE, whereas HPV vaccine and influenza vaccine slightly related to the risk of SLE.

The polling analysis showed no obvious association between vaccinations and the risk of SLE in our study, which differed from the previous review. The previous study included 12 studies with 8,732,085 participants [28–39], while our study included 17 studies with 45,067,349 individuals. We added five studies with an increase of 36,335,264 people providing a comprehensive assessment of the relationship between vaccinations and risk of SLE [16–19, 27]. In the added five studies, three mentioned the HPV vaccine [17–19] and two mentioned the COVID-19 vaccine [16, 27], with all cohort studies. The conclusions about HBV vaccine, HPV vaccine and influenza vaccine were consistent with the previous review, because the same included studies of HBV and influenza vaccine, and the additional three studies on HPV vaccine also support the conclusions of the previous study. There was no association between vaccinations and the risk of SLE was confirmed in different regions, whether it’s in North America, Europe or Asia. The prior review showed at an increased SLE risk with short vaccination times in five included trails but did not a specify time frame [28, 32, 35, 36, 38]. Interestingly, in our study, we found no significant association between the timing of vaccination, whether within or beyond 180 days, and an increased risk of SLE. Furthermore, in our subgroup analyses, case-control studies also demonstrated a significant association between vaccinations and an elevated SLE risk with the same included studies, while cohort studies indicated a marginally significant association.

The both studies showed that HBV vaccination was significantly associated with increased risk of SLE, because the both studies included the same trails. It has been reported that hepatitis B is contribute to onset or exacerbation of autoimmune disorders via molecular mimicry [40, 41]. Whereas, it has been proven the safety and efficacy in preventing HBV infection [42, 43], and ruled out any confirmed evidence that HBV vaccine causes autoimmune disorders [44]. HBV vaccine contains yeast, aluminium, thimerosal, hepatitis B surface antigen epitopes, and adjuvants, which may result in hepatitis B vaccine being associated with autoimmune diseases among susceptible adult vaccine recipients [44]. HBV vaccination induces autoimmunity and the possible mechanisms seem to be molecular mimicry, the production of particular autoantibodies, and the role of certain vaccine adjuvants. HBV vaccine accelerates SLE-like disease in an autoimmune genetically prone mouse model, the level of anti-dsDNA antibodies and resulted in early onset of proteinuria, and might increase risk of autoimmunity in genetically susceptible individuals [45, 46]. The result showed that HBV vaccination was significantly associated with increased risk of SLE, however, the included studies were observed 1–2 decades ago, with the development of vaccine technology, new clinical trials are needed to provide new evidence.

In the two studies, the most of observational studies have been on the HPV vaccine, HPV vaccine was demonstrated to be safe, well-tolerated, and highly efficacious in preventing persistent infections and cervical diseases among young women [47]. The epidemiological and molecular data suggest that the vast molecular homology between viral peptides and human proteins may involvement of HPV infection in the pathogenesis of SLE [48]. While, the adjuvants component of the HPV vaccine have been hypothesized to induce abnormal activation of the immune system. This may be resolve by advances in vaccine design and preparation processes, as well as improvements in adjuvants [49]. There is a female predominance for SLE, and occurs in middle-aged adults in all nationalities. There are significant similar in HPV vaccination populations and ages. The previous review and our study showed that HPV vaccination not related to increased risk of SLE, which is a benefit for females.

Viral infection is believed to modify the induction and development of autoimmune diseases, and triggered mechanisms including molecular mimicry, bystander activation, and immortalisation of infected B cells [50, 51]. The COVID-19 infectious disease was first emerged in 2019, which is caused by severe acute respiratory syndrome coronavirus 2 (SARS-CoV-2). Multiple autoantibodies and cytokine have been detected among SARS-CoV-2 infected patients including anti-CCP antibodies, anti-nuclear antibodies, majorly interleukin, tumour necrosis factor-α, and SARS-CoV-2 infection also diminished and dysfunctional T-regulatory cells [52–55]. Although widespread COVID-19 vaccine has reduced disease severity and mortality, vaccine-related adverse events such as autoimmune and autoinflammatory diseases have been documented, however these reports were case reports and there are no systematic large sample observational studies [56–60]. There was a study showed COVID-19 vaccination had a significant protective effect among all patients with SLE with COVID-19 [61], a non-causal association between of COVID-19 and risk of SLE [62], and COVID-19 vaccination reduced the risk of COVID-19-associated autoimmune diseases [16, 27]. In this study, the pooled findings showed that COVID-19 vaccination no associate with the risk of SLE. It seem that COVID-19 vaccine reduced the risk of autoimmune diseases by reducing viral infection. However, with only two studies, more cases and longer follow-up time clinical studies need to provide in the future to confirm our results.

The epidemiology of SLE is distributed unequally among geographical regions. SLE occurs more frequently in high-income countries. The USA, Poland, and Barbados had the highest SLE incidence. The United Arab Emirates, Barbados and Brazil had the highest SLE prevalence [63]. A previous systematic review showed that the highest incidence and prevalence of SLE were possibly in North America, and Asian people to have intermediate incidence and prevalence of SLE [64], for differences in gene environment interactions. Therefore, we employed the subgroup analysis by study design to observe the relationship between SLE and vaccination, and to observe whether vaccination is a potential pathogenesis of the different SLE incidence in different regions. However, in subgroup analysis showed that no obvious increased SLE risk associated with vaccine was observed in North America, Europe and Asia.

Studies have explored the link between vaccines and autoimmune diseases, revealing that microbial elements resembling human proteins might not trigger an immune response due to our immune systemic inherent tolerance mechanisms [65]. However, when these elements are encountered in the presence of impaired tolerance, autoimmunity can be induced. Factors like genetics and the environment play a role in modulating immune tolerance. Only a minority of vaccinated individuals develop autoimmune phenomena, indicating a genetic predisposition to vaccine-induced autoimmunity. Vaccines can activate the adaptive immune response to provide protection but might also stimulate hyperinflammatory conditions and produce specific autoantibodies, leading to adverse events. These reactions result from the interplay between the vaccinated personal susceptibility and vaccine components. Immune cross-reactivity, where certain vaccine components resemble specific human proteins, can cause the immune system to attack similar proteins in susceptible individuals, leading to autoimmune diseases-a phenomenon known as molecular mimicry [66]. In the case of HBV, influenza, and HPV vaccines, suspicions arise regarding their potential to trigger autoimmunity through molecular mimicry induced by viral particle vaccination [40, 67]. mRNA vaccines, such as the COVID-19 mRNA vaccine, act as both antigen and adjuvant, recognized by endosomal Toll-like receptors and cytosolic inflammasome components, thereby triggering inflammation and immunoreaction [68]. Studies have shown that the side effects of COVID-19 vaccines result from a temporary increase in IFN-I production alongside the induction of an immune response [69]. Adjuvants, which are compounds used to boost the immune response, can potentially disrupt immune tolerance. Vaccine adjuvants may enhance vaccine immunogenicity by activating the NLR pyrin domain-containing 3 (NLRP3) inflammasome [70]. The NLRP3 inflammasome plays a crucial role in both the innate and adaptive immune systems and is linked to various autoimmune diseases, including rheumatoid arthritis and systemic lupus erythematosus (SLE) [71]. IgE-mediated reactions, linked to polyethylene glycols as a known culprit, could be responsible for anaphylactic reactions following COVID-19 vaccination [72, 73]. Other components such as the buffering/oxidation inhibitor histidine and the non-ionic surfactant polysorbate 80 may also play a role in anaphylaxis or severe hypersensitivity reactions after vaccination [74]. Advancements in vaccine technology, increased attention to vaccine side effects and mechanisms, and the optimization of vaccination strategies may help explain why vaccination has not been associated with an increased risk of SLE.

### Implications and limitations

The main strength of our meta-analysis lies in the inclusion of 17 relevant observational studies, ensuring a robust evaluation of the association between vaccinations and the risk of SLE. With a substantial pooled sample size, we were able to effectively assess this relationship. Our findings suggest that vaccination may not pose a significant risk for SLE. Notably, we focused exclusively on cohort and case-control studies, effectively managing various confounding factors and enhancing the reliability of our conclusions. While our meta-analysis did not involve covariate analysis, the cohort studies we included adequately controlled for confounders, minimizing bias and ensuring the reliability of our findings.

Several limitations of the meta-analysis should be considered. First, subgroup analyses based on vaccine type would have been underpowered, the main reasons being limited vaccine studies. Additional well-designed studies with larger samples about various vaccine types are required to examine the association between vaccination and SLE. Second, the vaccination exposure window and follow-up time were inconsistent across the included studies. Third, all studies included in the meta-analysis were conducted in North America, Europe, and Asia, no studies were from African countries. Therefore, the findings of this meta-analysis cannot be generalised to African populations, additional observational studies from African countries are needed to provide epidemiological evidence for the influence of vaccinations on the risk of SLE in Africans.

## Conclusions

This meta-analysis indicates that vaccination may not be significantly associated with an increased risk of SLE. However, for HBV vaccination, it shows a potential association with a risk of SLE. Our meta-analysis results provide valuable insights, alleviating concerns about SLE risk post-vaccination and supporting further vaccine development efforts.

### Electronic supplementary material

Below is the link to the electronic supplementary material.

**Supplementary Material 1: Supplementary Fig. S1.** Sensitivity analysis of the risk of SLE caused by any vaccine

**Supplementary Material 2: Supplementary Tables, Supplementary Table 1-3:** Details of the Literature Search Strategy, **Supplementary Table 4:** Details of the excluded studies, **Supplementary Table 5:** Details of the NOS, **Supplementary Table 6:** Sensitivity analyses

## Data Availability

No datasets were generated or analysed during the current study.

## Abbreviations

SLE: systemic lupus erythematosus
PRISMA: preferred reporting items for systematic reviews and meta-analyses
PROSPERO: international prospective register of systematic reviews
MeSH: medical subject headings
OR: odds ratio
CI: confidence interval
NOS: Newcastle-Ottawa scale
ICD: international classification of diseases
COVID-19: coronavirus disease 2019
HPV: human papillomavirus
HBV: hepatitis B virus
HSV: herpes simplex virus
DTPP: diphtheria,tetanus,pertussis,poliomyelitis
NLRP3: NLR pyrin domain containing 3
