# Supplementary material for: Vaccination and the risk of systemic lupus erythematosus: a meta-analysis of observational studies
Source: Arthritis Res Ther. 2024 Mar 4;26:60. doi: 10.1186/s13075-024-03296-8 (PMC10910799; doi:10.1186/s13075-024-03296-8)
Supplement: Supplementary file 1 — Supplementary Material 1: Supplementary Fig. S1. Sensitivity analysis of the risk of SLE caused by any vaccine [file 13075_2024_3296_MOESM1_ESM.docx]

**Supplementary**

**Fig. S1.** Sensitivity analysis of the risk of SLE caused by any vaccine
