# Supplementary material for: Vaccination and the risk of systemic lupus erythematosus: a meta-analysis of observational studies
Source: Arthritis Res Ther. 2024 Mar 4;26:60. doi: 10.1186/s13075-024-03296-8 (PMC10910799; doi:10.1186/s13075-024-03296-8)
Supplement: Supplementary file 2 — Supplementary Material 2: Supplementary Tables, Supplementary Table 1-3: Details of the Literature Search Strategy, Supplementary Table 4: Details of the excluded studies, Supplementary Table 5: Details of the NOS, Supplementary Table 6: Sensitivity analyses [file 13075_2024_3296_MOESM2_ESM.doc]

**Supplementary**

**Table 1-3:** **Details of the Literature Search Strategy**

**Table 1** PubMed (September 3, 2023)

| Search | Query | Results |
| --- | --- | --- |
| 1 | (((("Lupus Erythematosus, Systemic"[MeSH Terms]) OR ("Systemic Lupus Erythematosus"[Title/Abstract])) OR ("Lupus Erythematosus Disseminatus"[Title/Abstract])) OR ("Libman Sacks Disease"[Title/Abstract])) OR ("SLE"[Title/Abstract]) | 86,744 |
| 2 | (("vaccination"[MeSH Terms]) OR ("Vaccination*"[Title/Abstract])) OR ("Active Immunization*"[Title/Abstract]) | 234,212 |
| 3 | ((((("Lupus Erythematosus, Systemic"[MeSH Terms]) OR ("Systemic Lupus Erythematosus"[Title/Abstract])) OR ("Lupus Erythematosus Disseminatus"[Title/Abstract])) OR ("Libman Sacks Disease"[Title/Abstract])) OR ("SLE"[Title/Abstract])) AND ((("vaccination"[MeSH Terms]) OR ("Vaccination*"[Title/Abstract])) OR ("Active Immunization*"[Title/Abstract])) | 659 |
| 4 | ("risk"[MeSH Terms]) OR (risk [Title/Abstract]) | 3,284,924 |
| 5 | (((((("Lupus Erythematosus, Systemic"[MeSH Terms]) OR ("Systemic Lupus Erythematosus"[Title/Abstract])) OR ("Lupus Erythematosus Disseminatus"[Title/Abstract])) OR ("Libman Sacks Disease"[Title/Abstract])) OR ("SLE"[Title/Abstract])) AND ((("vaccination"[MeSH Terms]) OR ("Vaccination*"[Title/Abstract])) OR ("Active Immunization*"[Title/Abstract]))) AND (("risk"[MeSH Terms]) OR (risk [Title/Abstract])) | 210 |

**Table 2** Cochrane Library (September 3, 2023)

| **Search** | **Query** | **Results** |
| --- | --- | --- |
| #1 | MeSH descriptor: [Lupus Erythematosus, Systemic] explode all trees | 1,444 |
| #2 | (Systemic Lupus Erythematosus): ti,ab,kw | 2.916 |
| #3 | (Lupus Erythematosus Disseminatus): ti,ab,kw | 0 |
| #4 | (Libman Sacks Disease): ti,ab,kw | 2 |
| #5 | (SLE): ti,ab,kw | 2345 |
| #6 | #1 OR #2 OR #3 OR #4 OR #5 | 3533 |
| #7 | MeSH descriptor: [Vaccines] in all MeSH products | 16038 |
| #8 | (Vaccination*): ti,ab,kw | 18,674 |
| #9 | (Active Immunization*): ti,ab,kw | 1,800 |
| #10 | #7 OR #8 OR #9 | 25,619 |
| #11 | #6 AND #10 | 79 |
| #12 | MeSH descriptor: [Risk] explode all trees | 58,451 |
| #13 | (risk): ti,ab,kw | 296,822 |
| #14 | #12 OR #13 | 300,325 |
| #15 | #11 AND #14 | 20 |

**Table 3** Embase (September 3, 2023)

| **Search** | **Query** | **Items found** |
| --- | --- | --- |
| #1 | 'lupus erythematosus, systemic'/exp | 117,377 |
| #2 | 'systemic lupus erythematosus': ab,ti | 83711 |
| #3 | 'lupus erythematosus disseminatus': ab,ti | 225 |
| #4 | 'libman sacks disease': ab,ti | 19 |
| #5 | 'sle': ab,ti | 67988 |
| #6 | #1 OR #2 OR #3 OR #4 OR #5 | 137,626 |
| #7 | 'vaccination'/exp | 254,742 |
| #8 | 'vaccination*': ab,ti | 234,405 |
| #9 | 'active immunization*': ab,ti | 4158 |
| #10 | #7 OR #8 OR #9 | 333,544 |
| #11 | #6 AND #10 | 2007 |
| #12 | 'risk'/exp | 3,093,160 |
| #13 | 'risk': ab,ti | 3,977,554 |
| #14 | #12 OR #13 | 4,887,556 |
| #15 | #11 AND #14 | 790 |

Table 4: Details of the excluded studies

|  | Study | Year | Title | Reason for exclued |
| --- | --- | --- | --- | --- |
| 1 | Avina-Galindo, A. M. | 2021 | Risk of hospitalization, admission to intensive care and mortality due to COVID-19 in patients with rheumatic diseases: A population-based matched cohort study | Conference abstract |
| 2 | Barbar-Smiley, F. | 2018 | Nasopharyngeal pneumococcus colonization in patients with childhood onset systemic lupus erythematosus (cSLE) | Conference abstract |
| 3 | Barbar-Smiley, F. | 2020 | Nasopharyngeal pneumococcus colonization in patients with childhood onset systemic lupus erythematosus (CSLE) compared to healthy controls | Conference abstract |
| 4 | Barbar-Smiley, F. | 2018 | Immunogenicity of pneumococcal vaccination and impact on nasopharyngeal pneumococcus colonization in patients with childhood onset systemic lupus erythematosus (cSLE) | Conference abstract |
| 5 | Barbar-Smiley, F. | 2019 | Immunogenicity of pneumococcal vaccination and impact on nasopharyngeal pneumococcus colonization in patients with childhood onset systemic lupus erythematosus (cSLE) | Conference abstract |
| 6 | Barbhaiya, M. | 2021 | FLARES AFTER SARS-COV-2 VACCINATION IN PATIENTS WITH SYSTEMIC LUPUS ERYTHEMATOSUS | Conference abstract |
| 7 | Beaulieu, M. C. | 2022 | Patient Mobilization for Vaccine Access and Improved Care during the COVID Pandemic | Conference abstract |
| 8 | Chung, H. | 2023 | Risk of exacerbation of rheumatic disease after COVID-19 vaccination | Conference abstract |
| 9 | Clark, M. T. | 2022 | Improving Pneumococcal Vaccination in Patients with Childhood Systemic Lupus Erythematos us in a Pediatric Rheumatology Clinic | Conference abstract |
| 10 | Cota, M. A. | 2022 | Systemic Lupus Erythematosus (SLE) Flare Following Second Dose of mRNA COVID-19 Vaccine | Conference abstract |
| 11 | Curtis, J. R. | 2014 | Herpes zoster infection across auto-immune and inflammatory diseases: Implications for vaccination | Conference abstract |
| 12 | Desai, N. | 2019 | Improving rates of cervical cancer screening and HPV vaccination in patients with lupus | Conference abstract |
| 13 | Desai, N. | 2019 | Improving rates of cervical cancer screening and prevention in patients with lupus | Conference abstract |
| 14 | Dhar, J. P. | 2017 | Lack of uptake of prophylactic human papilloma virus (HPV) vaccination among women with SLE in saginaw valley, a high risk population | Conference abstract |
| 15 | Dhillon, I. | 2023 | New-onset Immune-Mediated Disease Following SARS-CoV-2 Vaccination: A Case Series | Conference abstract |
| 16 | Dias, S. E. B. | 2023 | EVALUATION OF DISEASE ACTIVITY IN PATIENTS WITH SYSTEMIC LUPUS ERYTHEMATOSUS IN FOLLOW-UPATA UNIVERSITY HOSPITAL IN MANAUS AFTERVACCINATION AGAINST COVID-19 | Conference abstract |
| 17 | Ditto, M. C. | 2017 | Reasons why patients failed vaccinations vs influenza and Pneumococcus. Monocentric cross-sectional study | Conference abstract |
| 18 | Egbring, M. | 2009 | Risk of incident autoimmune diseases after hepatitis b vaccination: A large cohort study in the U.K. general practice research database | Conference abstract |
| 19 | Espanola, W. | 2023 | Risk factors for moderate to severe COVID-19 infection in systemic lupus erythematosus | Conference abstract |
| 20 | Feldman, C. | 2019 | Avoidable acute care use for vaccine-preventable illnesses among medicaid beneficiaries with lupus: Demographic and healthcare utilization differences | Conference abstract |
| 21 | Gerosa, M. | 2022 | THE IMPACT of ANTI-SARS-COV-2 VACCINES in A MULTICENTER COHORT STUDY of PATIENTS with SYSTEMIC LUPUS ERYTHEMATOSUS | Conference abstract |
| 22 | Gomez, G. | 2017 | Prevalence of pneumococcal vaccination in rheumatologic patients with community acquired pneumonia. Biobadasar registry | Conference abstract |
| 23 | Gomez-Puerta, J. A. | 2022 | Vaccination Against SARS-CoV2 in Patients with Systemic Autoimmune Diseases: A Safety Report from EULAR COVAX Registry | Conference abstract |
| 24 | Gonzalez, E. | 2017 | Immunogenicity of 13 valent pneumococcal vaccine in children with lupus: Single center experience in south Texas | Conference abstract |
| 25 | Grimaldi, L. | 2022 | Should we worry about flare-ups following vaccination in systemic lupus patients? A nationwide case-crossover study using the French healthcare database | Conference abstract |
| 26 | Grimaldi, L. | 2021 | Oral Presentation: Should We Worry about Flare-Ups Following Vaccination in Systemic Lupus Patients? A Nationwide Case-Crossover Study Using the French Healthcare Database | Conference abstract |
| 27 | Grimaldi-Bensouda, L. | 2012 | The risk of systemic lupus erythematosus associated with vaccines: A case-control study in france and canada | Conference abstract |
| 28 | Hamijoyo, L. | 2023 | Update on management of COVID-19 infection and vaccination in systemic lupus erythematosus | Conference abstract |
| 29 | Harifi, G. | 2020 | Vaccination in auto-immune and inflammatory rheumatic diseases AIIRD: The current practice among rheumatologists in the UAE | Conference abstract |
| 30 | Harris, J. | 2020 | Infl uenza Knowledge and Barriers to Vaccination in Immunosuppressed Patients in the Pediatric Rheumatology Clinic | Conference abstract |
| 31 | Kapoor, T. | 2016 | Herpetic viruses in lupus | Conference abstract |
| 32 | Kenninger, H. | 2020 | Determining the Zoster Vaccination Rate among Veterans on Chronic Immunosuppressive Therapy at the Southeast Louisiana Veterans Healthcare System - A Quality Indicator | Conference abstract |
| 33 | Bardenheier BH | 2016 | Anthrax Vaccine and the Risk of Rheumatoid Arthritis and Systemic Lupus Erythematosus in the U.S. Military: A Case–Control Study | The full text does not meet the inclusion criteria |
| 34 | C.C. Mok | 2018 | Long-term immunogenicity of a quadrivalent human papillomavirus vaccine in systemic lupus erythematosus | The full text does not meet the inclusion criteria |
| 35 | Mok CC | 2019 | Safety and immune response of a live-attenuated herpes zoster vaccine in patients with systemic lupus erythematosus: a randomised placebo-controlled trial | The full text does not meet the inclusion criteria |
| 36 | Chang CC | 2016 | Effects of annual influenza vaccination on morbidity and mortality in patients with Systemic Lupus Erythematosus: A Nationwide Cohort Study | The full text does not meet the inclusion criteria |
| 37 | Vista ES | 2012 | Influenza vaccination can induce new-onset anticardiolipins but not β2-glycoprotein-I antibodies among patients with systemic lupus erythematosus | The full text does not meet the inclusion criteria |
| 38 | So H | 2022 | So H, Li T, Chan V, Tam LS, Chan PK. Immunogenicity and safety of inactivated and mRNA COVID-19 vaccines in patients with systemic lupus erythematosus | The full text does not meet the inclusion criteria |
| 39 | Pope JE | 2004 | Close association of herpes zoster reactivation and systemic lupus erythematosus (SLE) diagnosis: case-control study of patients with SLE or noninflammatory musculoskeletal disorders | The full text does not meet the inclusion criteria |
| 40 | Guthridge JM | 2013 | Herpes zoster vaccination in SLE: a pilot study of immunogenicity | The full text does not meet the inclusion criteria |
| 41 | Wallin L | 2009 | Safety and efficiency of influenza vaccination in systemic lupus erythematosus patients | The full text does not meet the inclusion criteria |
| 42 | Stojanovich L | 2006 | Influenza vaccination of patients with systemic lupus erythematosus (SLE) and rheumatoid arthritis (RA) | The full text does not meet the inclusion criteria |
| 43 | Campos LM | 2013 | High disease activity: an independent factor for reduced immunogenicity of the pandemic influenza a vaccine in patients with juvenile systemic lupus erythematosus | The full text does not meet the inclusion criteria |
| 44 | Gorelik M | 2018 | Immunogenicity of sequential 13-valent conjugated and 23-valent unconjugated pneumococcal vaccines in a population of children with lupus | The full text does not meet the inclusion criteria |
| 45 | Krasselt M | 2021 | Humoral Immunity to Varicella Zoster Virus in Patients with Systemic Lupus Erythematosus and Rheumatoid Arthritis Compared to Healthy Controls | The full text does not meet the inclusion criteria |
| 46 | Petri M | 2023 | Effect of Systemic Lupus Erythematosus and Immunosuppressive Agents on COVID-19 Vaccination Antibody Response | The full text does not meet the inclusion criteria |
| 47 | Izmirly PM | 2022 | Evaluation of Immune Response and Disease Status in Systemic Lupus Erythematosus Patients Following SARS-CoV-2 Vaccination | The full text does not meet the inclusion criteria |
| 48 | Alyasin S | 2016 | Immunogenicity of 23-Valent Pneumococcal Vaccine in Children with Systemic Lupus Erythematosus | The full text does not meet the inclusion criteria |
| 49 | Grabar S | 2017 | Pneumococcal vaccination in patients with systemic lupus erythematosus: A multicenter placebo-controlled randomized double-blind study | The full text does not meet the inclusion criteria |
| 50 | Jiang X | 2023 | Risk of COVID-19 among unvaccinated and vaccinated patients with systemic lupus erythematosus: a general population study | The full text does not meet the inclusion criteria |
| 51 | Feldman CH | 2021 | Avoidable Acute Care Use for Vaccine-Preventable Illnesses Among Medicaid Beneficiaries With Lupus | The full text does not meet the inclusion criteria |
| 52 | Bartels LE | 2021 | Local and systemic reactogenicity of COVID-19 vaccine BNT162b2 in patients with systemic lupus erythematosus and rheumatoid arthritis | The full text does not meet the inclusion criteria |
| 53 | Chen J | 2023 | Varicella zoster virus reactivation following COVID-19 vaccination in patients with autoimmune inflammatory rheumatic diseases: A cross-sectional Chinese study of 318 cases | The full text does not meet the inclusion criteria |
| 54 | Chevet B | 2022 | COVID-19 Vaccine Uptake Among Patients With Systemic Lupus Erythematosus in the American Midwest: The Lupus Midwest Network (LUMEN) | The full text does not meet the inclusion criteria |
| 55 | Felten R | 2021 | Tolerance of COVID-19 vaccination in patients with systemic lupus erythematosus: the international VACOLUP study | The full text does not meet the inclusion criteria |
| 56 | Gerosa M | 2022 | The Impact of Anti-SARS-CoV-2 Vaccine in Patients with Systemic Lupus Erythematosus: A Multicentre Cohort Study | The full text does not meet the inclusion criteria |
| 57 | Angelo MG | 2014 | Pooled analysis of large and long-term safety data from the human papillomavirus-16/18-AS04-adjuvanted vaccine clinical trial programme | The full text does not meet the inclusion criteria |
| 58 | Leung J | 2022 | Leung J, Anderson TC, Dooling K, Xie F, Curtis JR. Recombinant Zoster Vaccine Uptake and Risk of Flares Among Older Adults With Immune-Mediated Inflammatory Diseases in the US | The full text does not meet the inclusion criteria |
| 59 | Esposito D | 2018 | Incidence of outcomes relevant to vaccine safety monitoring in a US commercially-insured population | The full text does not meet the inclusion criteria |
| 60 | Patel NJ | 2023 | Factors associated with COVID-19 breakthrough infection among vaccinated patients with rheumatic diseases: A cohort study | The full text does not meet the inclusion criteria |
| 61 | Geier DA | 2015 | A case-control study of quadrivalent human papillomavirus vaccine-associated autoimmune adverse events | The data from the same study |

**Table 5: Details of the NOS**

|  | **Study** | **Year** | **Selection** | **Comparability** | | **Outcome** | **Overall quality**  **score** |
| --- | --- | --- | --- | --- | --- | --- | --- |
| 1 | Ju HJ | 2023 | ★★★ |  | ★★ | | 5 |
| 2 | Peng K | 2023 | ★★ | ★★ | ★★★ | | 7 |
| 3 | Skufca J | 2018 | ★★ | ★ | ★★★ | | 6 |
| 4 | Hviid A | 2018 | ★★ |  | ★★★ | | 5 |
| 5 | Miranda S | 2017 | ★★ | ★ | ★★ | | 5 |
| 6 | Geier D | 2016 | ★★★ |  | ★★★ | | 6 |
| 7 | Bardenheier BH | 2016 | ★★ | ★★ | ★★★ | | 7 |
| 8 | Lai YC | 2015 | ★★★ |  | ★★ | | 5 |
| 9 | Grimaldi-Bensouda L | 2014 | ★★★ | ★★ | ★★ | | 7 |
| 10 | Persson I | 2014 | ★★★★ | ★★ | ★★★ | | 9 |
| 11 | Zou Y | 2014 | ★★★ | ★★ | ★★ | | 7 |
| 12 | Angelo MG | 2014 | ★★★ | ★ | ★★ | | 6 |
| 13 | Arnheim-Dahlstrom L | 2013 | ★★★★ | ★★ | ★★ | | 8 |
| 14 | Chao C | 2012 | ★★★ | ★ | ★★ | | 6 |
| 15 | Verstraeten T | 2008 | ★★★ | ★ | ★★ | | 6 |
| 16 | Geier DA | 2005 | ★★★ | ★ | ★★ | | 6 |
| 17 | Cooper GS | 2002 | ★★ | ★★ | ★★★ | | 7 |

**Table 6: Sensitivity analyses**

|  |  | **OR** | **95% CI** | **I^2^/%** | ***P* value** |
| --- | --- | --- | --- | --- | --- |
|  | **Total** | 1.14 | 0.86-1.52 | 78.1 | 0.348 |
|  | **Excluded study** |  |  |  |  |
| 1 | Ju HJ 2023 | 1.21 | 0.88-1.66 | 78.3 | 0.237 |
| 2 | Peng K 2023 | 1.24 | 0.97-1.59 | 68.6 | 0.085 |
| 3 | Skufca J 2018 | 1.128 | 0.85-1.50 | 79.2 | 0.411 |
| 4 | Hviid A 2018 | 1.19 | 0.88-1.60 | 79.3 | 0.266 |
| 5 | Miranda S 2017 | 1.01 | 0.79-1.30 | 70.8 | 0.92 |
| 6 | Geier DA 2017 | 1.17 | 0.86-1.59 | 79.5 | 0.321 |
| 7 | Bardenheier BH 2016 | 1.16 | 0.87-1.54 | 79.5 | 0.326 |
| 8 | Lai YC 2015 | 1.17 | 0.87-1.56 | 79.4 | 0.299 |
| 9 | Grimaldi-Bensouda L 2014 | 1.17 | 0.87-1.58 | 79.5 | 0.299 |
| 10 | Persson I 2014 | 1.21 | 0.85-1.71 | 79.5 | 0.292 |
| 11 | Zou Y 2014 | 1.07 | 0.81-1.42 | 75.9 | 0.629 |
| 12 | Angelo MG 2014 | 1.14 | 0.86-1.52 | 79.5 | 0.359 |
| 13 | Arnheim-Dahlström L 2013 | 1.13 | 0.84-1.52 | 79.2 | 0.401 |
| 14 | Chao C 2012 | 1.16 | 0.85-1.57 | 79.4 | 0.339 |
| 15 | Verstraeten T 2008 | 1.13 | 0.85-1.50 | 79.2 | 0.400 |
| 16 | GEIER DA 2005 | 1.11 | 0.83-1.43 | 77.6 | 0.536 |
| 17 | Cooper GS 2002 | 1.13 | 0.84-1.52 | 78.6 | 0.428 |
